# Change in blood pressure status defined by 2017 ACC/AHA hypertension guideline and risk of cardiovascular disease: results of over a decade of follow-up of the Iranian population

**DOI:** 10.3389/fcvm.2023.1044638

**Published:** 2023-06-09

**Authors:** Maryam Kabootari, Seyed Saeed Tamehri Zadeh, Mitra Hasheminia, Fereidoun Azizi, Farzad Hadaegh

**Affiliations:** ^1^ Metabolic Disorders Research Center, Golestan University of Medical Sciences, Gorgan, Iran; ^2^ Prevention of Metabolic Disorders Research Center, Research Institute for Endocrine Sciences, Shahid Beheshti University of Medical Sciences, Tehran, Iran; ^3^ Department of Biostatistics and Epidemiology, Research Institute for Endocrine Sciences, Shahid Beheshti University of Medical Sciences, Tehran, Iran; ^4^ Endocrine Research Center, Research Institute for Endocrine Sciences, Shahid Beheshti University of Medical Sciences, Tehran, Iran

**Keywords:** elevated blood pressure, stage 1 hypertension, cardiovascular disease, 2017 ACC/AHA hypertension guideline, changes in the BP category

## Abstract

**Background:**

Hypertension (HTN) is known to be the leading cause of cardiovascular disease (CVD) and mortality. We aimed to assess the impact of changes in 3 years in different blood pressure (BP) categories on incident CVD.

**Methods:**

In this study, 3,685 Tehranians aged ≥30 years (42.2% men) free of prevalent CVD with BP level <140/90 mmHg and not on BP-lowering medications were enrolled. Participants were grouped according to baseline BP category using the 2017 ACC/AHA hypertension guideline definition: normal BP (<120/80 mmHg), elevated BP (120–129/<80), and stage 1 HTN (130–139 and/or 80–89). The hazard ratio of incident CVD by changes in the BP category was estimated after adjustment for traditional risk factors using Cox's proportional hazard model, with stable normotension as a reference.

**Results:**

During a median follow-up of 11.7 years, 346 CVD events (men = 208) occurred. Compared to the reference group, among participants with normal BP at baseline, only those with BP rising to stage 1 HTN [1.47 (0.99–2.16)], and among those with stage 1 HTN at baseline, regression to elevated BP [1.80 (1.11–2.91)], remaining at stage 1 [1.80 (1.29–2.52)], and progression to stage 2 HTN [1.81 (1.25–2.61)] had a higher risk for CVD; however, regression to normal BP attenuated this risk [1.36 (0.88–2.12)]. Conversion from elevated BP to any other categories had no significant association with CVD risk.

**Conclusions:**

Generally, prevalent stage 1 HTN (regardless of changing category) and incident stage 1 HTN were significantly associated with a higher risk of CVD; even regression to elevated BP did not attenuate the risk. Accordingly, these populations are potential candidates for antihypertensive management.

## Introduction

Cardiovascular disease (CVD) is the leading cause of death and the major contributive cause of disability among the Iranian population. Studies from different main cities in Iran demonstrated that the crude incidence rate of CVD is between 10 and 13 per 1,000 person-years (1, 2). Hypertension (HTN) is one of the main contributive factors for CVD and mortality (3, 4). Near 80% of the attributable burden of HTN is in low- and middle-income countries, especially in the Middle East and North Africa (MENA) region (5). In the MENA region, the prevalence of HTN is about 26.2% (6), and approximately 16.5% of all deaths and 6.1% of all disability-adjusted life years (DALYs) were attributable to HTN (5). Tehran is not exempt from the HTN epidemic and is confronted with a substantial burden, as a study demonstrated that HTN has a population-attributable fraction of 21% for CVD and 17% for all-cause mortality (7).

The 2017 American College of Cardiology/American Heart Association (ACC/AHA) HTN guideline lowered the HTN threshold from systolic blood pressure (SBP)/diastolic blood pressure (DBP) from ≥140/90 to ≥130–80 mmHg (8, 9). This new guideline defined new categories: elevated blood pressure (120–129/<80) and stage 1 HTN (130–139 and/or 80–89) (9).

A meta-analysis declared that pre-HTN status (120–140/80–90) is significantly associated with a higher risk of CVD events (10). Moreover, we showed that each year >5% of Iranian adults developed pre-HTN (11). Whether CVD risk among the pre-HTN population is attributable to the direct effect of pre-HTN or is mediated by conversion to HTN is a matter of debate.

The association between different categories of blood pressure (BP) using 2017 ACC/AHA and incident CVD has been evaluated in previous studies; however, the majority focused on snapshot measurement of those (12), and only a few studies have investigated the effect of change of BP category on incident CVD (13–16). A large population-based study among the Korean population revealed that compared to individuals who maintained normal BP, conversion from elevated BP to any other BP categories and even remaining in elevated BP was associated with a higher risk of CVD. This pattern was also found for stage 1 HTN (14).

In the current study, we aimed at assessing the association between change in BP category after 3 years of follow-up and incident CVD among Tehranian adults in the population-based cohort study, namely, the Tehran Lipid and Glucose Study (TLGS).

## Materials and methods

### Study population

The TLGS is an ongoing large prospective population-based study of a representative urban sample of the Tehran population investigating the risk factors and outcomes of noncommunicable disease.

In brief, participants have been recruited in the first (1999–2001) and second (2002–2005) phases with ongoing examinations at 3-year intervals. Details of study design, sampling frame, and rationale have been explained previously (17). In the current study, 7,123 individuals aged ≥30 years were included (all from the second phase). After excluding those with known (i.e., those taking BP-lowering medications at baseline visit) or newly diagnosed HTN (*n* = 1,817), prevalent CVD, or incident CVD before the second examination (*n* = 347), 4,959 individuals remained. Other exclusions were those with missing data on BP, age, gender, smoking, education, diabetes status, total cholesterol (TC), body mass index (BMI), family history (FH) of premature CVD, (*n* = 1,274, considering overlap features between numbers), leading to 3,685 participants with complete data (respondents) who were followed up until March 2018. According to the Helsinki Declaration guidelines, all participants provided written informed consent, and the local ethics committee approved the study (medical ethics committee of the Research Institute for Endocrine Sciences).

### Clinical and laboratory measurements

During interviews at baseline and each follow-up examination, demographic information, medical history, smoking, and history of CVD were obtained from participants using a pretested questionnaire. Anthropometric measurements [weight, height, and waist circumferences (WCs)] have been described elsewhere (17). BMI was measured as weight in kilograms divided by the square of height (m^2^). BP was measured twice on the right arm of participants in a seated position after at least 15 minutes of rest, using a standardized mercury sphygmomanometer (calibrated by the Iranian Institute of Standards and Industrial Researches). Then, the mean of two measurements was considered as the BP of the participants. Pulse rate was measured only one time by palpating the radial pulse for 30 s.

Blood samples were taken between 7:00 and 9:00 AM after 12–14 h overnight fasting. Details about measurements of serum glucose and TC have been previously reported (17).

### Definition of terms

For categorization of BP status, we used 2017 ACC/AHA guideline (9) criteria as follows: normal BP as SBP <120 and DBP <80, elevated BP as SBP 120–129 and DBP <80 mmHg, and stage 1 HTN as SBP 130–139 or DBP 80–89 mmHg at the first visit among those without a history of taking BP-lowering medications. Then, we checked for changes for each different category over the next examination. For those with normal BP at baseline, changes include remaining in normal BP, progression to elevated BP, stage 1 HTN, and stage 2 HTN (SBP ≥ 140 or DBP ≥ 90). For those with elevated BP, regression to normal BP, remaining in the previous status, and progression to stage 1 or 2 HTN were examined. Similarly, for those with stage 1 HTN, regression to normal BP and elevated BP, remaining in the previous status, and progression to stage 2 HTN were assessed.

We described type 2 diabetes as fasting plasma glucose (FPG) ≥7 mmol/L or taking antihyperglycemic drugs. Smoking was defined as the current usage of any kind of tobacco. Family history of premature CVD reflected a prior diagnosis of CVD in female first-degree relatives aged <65 years or male first-degree relatives aged <55 years. Educational status was categorized into three levels: less than 6 years, 6–12 years, and ≥12 years of formal education. Hypercholesterolemia was defined as TC levels ≥5.1 mmol/L or using lipid-lowering drugs. In the first examination of the study, we defined individuals participating in vigorous physical activity at least 3 days per week as physically active. Furthermore, for those who entered the second phase, being physically active was defined as achieving a minimum of at least 1,500 MET (metabolic equivalent task)-minutes (18) per week.

Pulse pressure (PP) was defined as SBP minus DBP.

### Definition of outcome

Coronary heart disease (CHD) included cases of (1) definite myocardial infarction (MI): diagnosed by electrocardiogram (ECG) and biomarkers; (2) probable MI: defined by ECG findings plus cardiac signs or symptoms and biomarkers indicating negative or equivocal results; (3) unstable angina pectoris: new cardiac symptoms or changing symptom patterns and positive ECG findings with normal biomarkers; (4) angiography proven CHD; and (5) CHD death (any death in hospital due to CHD according to the criteria mentioned above or sudden cardiac death caused by cardiac disease occurring ≤1 h after the beginning of symptoms based on verbal autopsy documents outside of the hospital). The present study described CVD as a composite measure of any CHD events, stroke, or cerebrovascular death. Details of the CVD outcomes have been published before (17).

### Statistical analysis

Differences in baseline characteristics between different categories of BP were evaluated using analysis of variance (ANOVA) and chi-square test as appropriate. To compare respondents with nonrespondents (those with missing data or without any follow-up after the second examination), the mean difference [95% confidence interval (CI)] of continuous variables and the mean differences in the prevalence (95% CI) of each category of categorical variables were estimated. We did not find any interaction between gender and different categories of BP change (all *P*-values >0.26); hence, the analysis was performed on the whole sample.

Multivariable Cox proportional hazard regression was used to assess the hazard ratios (HRs) of each BP category at baseline (elevated BP and stage 1 compared with normotension) as well as changing status and for CVD, considering those that remained in the normotension group as a reference. Time to event was defined by the time of censoring or the event occurring, whichever came first. We censored participants in the case of other causes of non-cardiovascular (non-CV) death, leaving the district, or being in the study until 20 March 2018, without any event.

Two models were defined: model 1 was adjusted for age and gender, and model 2 was further adjusted for baseline BMI, pulse rate, current smoking, education, low physical activity, TC, FH of premature CVD, hypercholesterolemia, and diabetes.

The proportionality in the Cox model was calculated with the Schoenfeld residual test; generally, all proportionality assumptions were appropriate. Statistical analysis was performed using STATA version 14 (Stata Corp LP, College Station, TX, United States) statistical software. *P*-values <0.05 were considered statistically significant.

## Results

A total of 3,685 participants (women = 57.8%) were enrolled. The mean (SD) of age and BMI among men were 46.4 (12.0) years and 26.4 (3.8) kg/m^2^, respectively. The corresponding values were 44.5 (10.3) years and 28.7 (4.4) kg/m^2^ for women. Moreover, 669 (31.4%) women were in post-menopausal state, and 100 women among them had CVD events during follow-up.

During a median (IQR) follow-up of 11.7 (10.8–12.6) years after the change in BP measurement, 346 CVD events (men = 208) occurred with an incidence rate of 10.0 (8.7–11.4) and 4.7 (3.9–5.5) per 1,000 persons-years for men and women, respectively.

The baseline characteristics of the participants across BP change categories are summarized in Table 1. Generally, there was a rising trend in almost all baseline characteristics among those with stage 1 HTN compared to those with elevated and normal BP at the baseline visit. Moreover, there was a significant difference between groups in baseline characteristics except for current smoking, low physical activity, and FH of premature CVD.

**Table 1:** Baseline characteristics of the participants across BP change categories.

	NBP-NBP (*N* = 1,672)	NBP-elevated (*N* = 136)	NBP-HTN1 (*N* = 374)	NBP-HTN2 (*N* = 71)	E-NL (*N* = 133)	E-E (*N* = 65)	E-HTN1 (*N* = 108)	E-HTN2 (*N* = 46)	HTN1-NL (*N* = 312)	HTN1-E (*N* = 119)	HTN1-HTN1 (*N* = 417)	HTN1-HTN2 (*N* = 232)
Continuous variables												
Age (y)	42.0 (9.6)	49.7 (12.4)	44.6 (10.1)	48.9 (12.0)	48.6 (12.3)	51.8 (12.8)	51.3 (12.7)	55.1 (10.1)	43.9 (9.3)	51.9 (10.9)	47.0 (11.0)	53.5 (11.1)
BMI (kg/m^2^)	26.8 (4.0)	27.1 (5.0)	27.8 (4.4)	28.6 (4.2)	27.5 (4.4)	27.6 (4.2)	28.7 (4.2)	28.1 (4.4)	28.6 (4.7)	29.9 (4.3)	29.1 (4.1)	28.8 (4.2)
SBP (mmHg)	103.8 (8.3)	110.6 (5.8)	108.1 (6.8)	110.9 (6.8)	123.4 (2.7)	123.5 (2.5)	123.5 (2.8)	124.1 (3.0)	117.2 (9.5)	126.1 (8.8)	123.1 (8.9)	127.6 (8.5)
DBP (mmHg)	68.2 (6.2)	69.3 (6.2)	71.3 (5.3)	72.2 (5.8)	73.1 (4.8)	71.9 (5.6)	74.2 (3.8)	73.1 (6.8)	81.7 (4.1)	81.0 (6.2)	82.7 (3.8)	81.9 (5.3)
Pulse rate (beats/min)	80.5 (10.8)	79.4 (11.3)	81.2 (10.9)	78.8 (12.0)	80.6 (10.5)	78.1 (11.2)	78.5 (11.0)	79.3 (11.9)	81.6 (10.5)	82.0 (11.3)	81.1 (10.7)	80.8 (10.9)
Pulse pressure (mmHg)	35.6 (7.4)	41.3 (7.8)	36.8 (6.9)	38.8 (7.3)	50.3 (5.7)	51.6 (5.9)	49.3 (4.5)	51.0 (5.9)	35.4 (10.9)	45.0 (11.9)	40.4 (10.1)	45.7 (11.0)
FPG (mmol/L)	5.1 (1.2)	5.4 (1.8)	5.2 (1.2)	5.8 (2.2)	5.4 (1.8)	5.7 (1.9)	5.9 (1.8)	5.7 (1.1)	5.4 (1.5)	5.9 (1.8)	5.5 (1.6)	5.9 (2.1)
2 h-PCPG (mmol/L)	6.0 (2.3)	6.6 (2.9)	6.2 (2.6)	7.1 (3.9)	6.7 (3.0)	6.8 (3.5)	7.9 (4.4)	7.9 (3.4)	6.8 (2.9)	7.2 (3.9)	6.9 (2.8)	7.4 (3.1)
TC (mmol/L)	4.9 (1.0)	5.2 (0.94)	5.03 (0.98)	5.3 (1.1)	5.3 (1.0)	5.2 (1.1)	5.0 (0.9)	5.3 (1.1)	5.2 (0.9)	5.4 (1.1)	5.2 (1.0)	5.2 (1.0)
Categorical variables												
Gender (male)	617 (37)	64 (47.1)	168 (44.9)	26 (36.6)	63 (47.4)	35 (53.8)	57 (52.8)	23 (50.0)	117 (37.5)	48 (40.3)	225 (54.0)	113 (48.7)
Hypercholesterolemia	632 (37.18)	69 (50.7)	157 (42.0)	37 (52.1)	70 (52.6)	36 (55.4)	44 (40.7)	22 (47.8)	160 (51.3)	63 (52.9)	195 (46.8)	124 (53.4)
Lipid-lowering drugs	21 (1.3)	4 (2.9)	10 (2.7)	1 (1.4)	6 (4.5)	3 (4.6)	3 (2.8)	4 (8.7)	11 (3.5)	3 (2.5)	18 (4.3)	6 (2.6)
Diabetes	69 (4.1)	10 (7.4)	17 (4.5)	11 (15.5)	11 (8.3)	9 (13.8)	20 (18.5)	8 (17.4)	23 (7.4)	15 (12.6)	32 (7.7)	37 (15.9)
Glucose-lowering drugs	31 (1.9)	6 (4.4)	12 (3.2)	7 (9.9)	6 (4.5)	3 (4.6)	8 (7.4)	2 (4.3)	10 (3.2)	5 (4.2)	15 (3.6)	19 (8.2)
Current smoking	245 (14.7)	23 (16.9)	46 (12.3)	7 (9.9)	13 (9.8)	11 (16.9)	12 (11.1)	4 (8.7)	40 (12.8)	14 (11.8)	58 (13.9)	19 (8.2)
FH of CVD	308 (18.4)	34 (25.0)	76 (20.3)	11 (15.5)	20 (15.0)	13 (20.0)	16 (14.8)	7 (15.2)	65 (20.8)	20 (16.8)	73 (17.5)	41 (17.7)
Low PH/A	1,051 (62.9)	78 (57.4)	245 (65.5)	47 (66.2)	79 (59.4)	39 (60.0)	54 (50.0)	26 (56.5)	193 (61.9)	76 (63.9)	263 (63.1)	152 (65.5)
Education, years												
<6	365 (21.8)	57 (41.9)	105 (28.1)	29 (40.8)	54 (40.6)	28 (43.1)	44 (40.7)	26 (56.5)	84 (26.9)	66 (55.5)	144 (34.5)	109 (47.0)
6–12	1,013 (60.6)	61 (44.9)	218 (58.3)	34 (47.9)	64 (48.1)	30 (46.2)	48 (44.4)	15 (32.6)	180 (57.7)	43 (36.1)	209 (50.1)	96 (41.1)
>12	294 (17.6)	18 (13.2)	51 (13.6)	8 (11.3)	15 (11.3)	7 (10.8)	16 (14.8)	5 (10.9)	48 (15.4)	10 (8.4)	64 (15.3)	27 (11.6)

BP, blood pressure; HTN, hypertension; BMI, body mass index; SBP, systolic blood pressure; DBP, diastolic blood pressure; TC, total cholesterol; CVD, cardiovascular disease; NBP, normal blood pressure; E-NL, elevated-normal; E-E, elevated-elevated; PSPG, postchallenge plasma glucose; PH/A, physical activity; FPG, fasting plasma glucose; FH, family history.

Furthermore, as shown in Supplementary Table S1, baseline characteristics of the respondent and nonrespondent groups were almost similar except that respondents were more likely to be men and had lower levels of BMI and higher prevalence of diabetes, glucose-lowering medication usage, and smoking compared with nonrespondents.

As displayed in Table 2, we assessed the association between elevated BP and stage 1 HTN and incident CVD. Considering normal BP as reference, elevated BP did not have any significant association with incident CVD in both models. However, stage 1 HTN increased the risk of CVD in model 1 [HR = 1.69 (1.35–2.12)] as well as model 2 [HR = 1.55 (1.23–1.96)].

**Table 2:** Multivariate HR and 95% CI of incident CVD for elevated BP and stage 1 HTN at baseline according to 2017 ACC/AHA hypertension guideline.

	** **	Model 1		Model 2	
	E/N	HR (95% CI)	*p*-value	HR (95% CI)	*p*-value
Normal	150/2,253	**Reference**	** **	**Reference**	
Elevated BP	37/352	0.92 (0.64–1.33)	0.664	0.86 (0.59–1.24)	0.421
Stage 1 HTN	159/1,080	**1.69** **(1.35–2.12)**	**<0.001**	**1.55** **(1.23–1.96)**	**<0.001**
Age, years	** **	**1.06** **(1.04–1.06)**	**<0.001**	**1.06** (**1.05–1.07)**	**<0**.**001**
Women (men as reference)		0.88 (0.68–1.15)	0.349	**0.51** (**0.39–0.66)**	**<0**.**001**
BMI, kg/m^2^				1.01 (0.99–1.04)	<0.35
Hypercholesterolemia, yes				**1.64** (**1.32–2.05)**	**<0**.**001**
Diabetes, yes				**2.32** (**1.76–3.07)**	**<0**.**001**
Current smoking, yes				**1.84** (**1.38–2.45)**	**0**.**001**
Education level, years					
<6				**Reference**	
6–12				1.19 (0.82–1.73	0.348
≥12				1.26 (0.84–1.88)	0.258
Low physical activity, yes				1.10 (0.88–1.37)	0.411
FH of CVD, yes				**1.39** (**1.07–1.81)**	**0**.**013**
Pulse rate				1.00 (0.99–1.01)	0.937

BMI, body mass index; CVD, cardiovascular disease; HRs, hazard ratios; CI, confidence interval; BP, blood pressure; HTN, hypertension; ACC/AHA, American College of Cardiology/American Heart Association; FH, family history.

Model 1 was adjusted for age and sex. Model 2 was adjusted for age, sex, BMI, pulse rate, smoking, education, physical activity, FH of premature cardiovascular disease, hypercholesterolemia, and diabetes.

Bold values mean that they are statistically significant.

**Figure 1:**
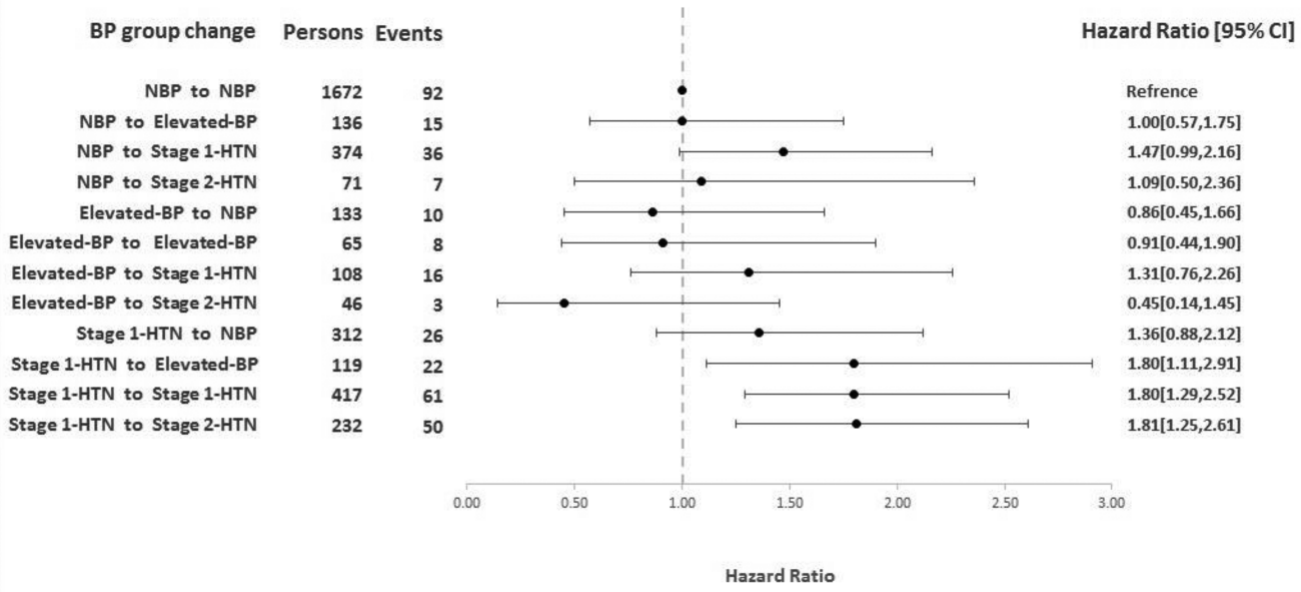
HRs (95% CI) of CVD for change in BP categories between the baseline visit and first follow-up according to 2017 ACC/AHA hypertension guideline adjusted for age, gender, BMI, pulse rate, smoking, education, physical activity, FH of premature cardiovascular disease, hypercholesterolemia, and diabetes. HR, hazard ratio; CI, confidence interval; BP, blood pressure; ACC/AHA, American College of Cardiology/American Heart Association; BMI, body mass index; FH, family history.

Figure 1 and Supplementary Table S2 summarize the HRs of CVD for change in BP categories between the baseline visit and the first follow-up in the two models. Among participants with normal BP at the baseline visit, only those with BP rising to HTN stage 1 had a higher risk for CVD [HR (95% CI): 1.43 (0.97–2.10), *P* = 0.07] in model 1 and model 2 [1.47 (0.99–2.16)]. Regarding those with elevated BP at baseline examination, regression to normal BP, remaining in the previous status, or progression to stages 1 and 2 HTN′ had a virtually similar risk of CVD as those with sustained normal BP (reference group). Comparing those with stage 1 HTN at baseline visit to the reference group, even regression to elevated BP [1.80 (1.11–2.91)] or remaining in the stage 1 HTN [1.80 (1.29–2.52)], as well as progression stage 2 HTN [1.81 (1.25–2.61)], were all associated with a significantly higher risk of CVD in the full adjusted model.

As a sensitivity analysis, we also examined the impact of change in PP on CVD outcomes, since baseline PP was found to be an important indicator of CVD events (19, 20). Regarding change in PP, when we categorized PP changes as quartiles (considering first quartile as the reference), we found no significant association between change in PP and risk of CVD events (Supplementary Table S3).

## Discussion

The current study assessed the impact of a 3-year change in the BP category using the 2017 ACC/AHA HTN definition on incident CVD. First, we detected that in contrast to elevated BP, stage 1 HTN at baseline increased the risk of CVD. Second, we found that newly developed stage 1 HTN is associated with a 47% higher risk of developing CVD. Moreover, among those with stage 1 HTN at baseline, whether remaining at the same stage or regression to elevated BP had about an 80% higher risk of developing CVD events independent of well-known CVD risk factors.

To the best of our knowledge, only a few studies conducted in the East Asian populations (13–16, 21) examined the association between the change of BP category using ACC/AHA 2017 HTN guideline and CVD. Son et al. designed a study to determine the association between change in BP within 2 years and incident CVD in individuals with normal BP. They revealed that compared to those who maintained normal BP, participants who progressed to elevated BP and stage 1 HTN had a 26% and 23% significantly higher risk for CVD, respectively. Additionally, the authors stratified patients with stage 1 HTN into three groups based on the 2017 ACC/AHA definition: stage 1 isolated systolic hypertension (ISH), isolated diastolic hypertension (IDH), and systolic diastolic hypertension (SDH), and found that BP elevation to stage 1 ISH and SDH, but not IDH, significantly increased the CVD risk (21). In our study, we found out that progressing from normal BP to stage 1 HTN was significantly associated with a 47% higher risk of CVD, while those who converted from elevated BP to stage 1 HTN had about 30% increased risk of HTN that did not reach to the significant level. As Whelton et al. demonstrated, for every 10 mm Hg increase in SBP, 53% higher risk of atherosclerotic cardiovascular disease can be found (22). Therefore, it is plausible that we expect worsened outcomes for those whose BP progresses from normal to stage 1 HTN relative to those who progress from elevated BP to stage 1 HTN. Another study that was carried out by Lee et al. among over 6 million Korean adults aged 20–39 years demonstrated that among those with stage 1 ISH, IDH, and SDH, regression to normal and elevated BP, remaining in the previous state, change to another stage 1 subgroups, and progression to stage 2 HTN were all associated with a significantly higher risk of CVD (14). Similarly, in the current study, among participants with stage 1, remaining in stage 1, progressing to stage 2, and even regression to elevated BP were significantly associated with about 80% higher risk of CVD.

In line with our findings, a Chinese study reported that compared to those who maintained BP <130/80 mmHg, remaining in stage 1, and progression to stage 2 HTN increased the CVD risk (16). Moreover, another large Chinese cohort considered those with regression from stage 1 HTN at baseline to normal BP after 4 years of follow-up as a reference and detected a higher risk of CVD events in those who remained at stage 1 HTN and progressed to stage 2 HTN but not those regressed to elevated BP (13).

Our study contains some strengths that are worth acknowledging. First, as far as we are aware, only a few studies have examined the association between change in the BP category based on the 2017 ACC/AHA definition and CVD risk. Second, the current study is a relatively large population-based prospective study with nearly a decade of follow-up. Another strength of our study is that we enrolled young, middle-aged, and elderly individuals; therefore, our results can be extrapolated to all Iranian urban adult population. Fourth, as recommended by the 2017 ACC/AHA BP guideline, we measured participants’ BP twice and determined their BP status based on the average of two readings (14).

Our study also has some limitations. First, previous studies have emphasized that the SBP alone is an important contributor for CVD events (23, 24). Using JNC VII definition of HTN, we had previously showed that isolated systolic hypertension (SBP ≥ 140 and DBP < 90) increased the risk of CVD (24). In the current study, we had only 130 participants with stage 1 ISH using 2017 ACC/AHA guideline; hence, due to the limited statistical power, we were not able to examine the impact of changing status of ISH on CVD. Second, the results could not be reported as gender stratified, especially as reported by Kringeland et al., stage 1 HTN was a stronger risk factor for acute coronary syndrome (ACS) during midlife in women than in men (25). Moreover, in the current study, we found that the incidence rate of CVD events was reported to be twofold higher among men than women, and similarly, the higher rate of CVD and CVD mortality in men compared to women was reported in other cohorts of the country; however, this disparity in CVD events was more prominent in the capital and the issue that might be related to the impact of social determinant of health such as the impact of air pollution, psychological stress, economical stress, and job stress affected the Tehranian men (R). However, we did not find any interaction between gender and different categories of BP change the issue that might be related to the power of study. Third, data about diet, especially salt intake, and environmental factors, including air pollution, were not available at the baseline recruitment of the study. We previously showed that air pollution has a major effect on incident HTN (26). Fourth, adjustment for changes of potential confounders over time was not performed. Finally, this study was performed in an Iranian urban population, and further research is necessary to determine whether the results could be generalized to other populations.

## Conclusion

Generally, prevalent stage 1 HTN regardless of changing category and incident stage 1 HTN were significantly associated with a higher risk of CVD; moreover, even regression to elevated BP did not attenuate the risk. Accordingly, these populations are potential candidates for antihypertensive management to prevent or at least delay the development of CVD events.

## Data Availability

The raw data supporting the conclusions of this article will be made available by the authors, without undue reservation.
