# Supplementary material for: Change in blood pressure status defined by 2017 ACC/AHA hypertension guideline and risk of cardiovascular disease: results of over a decade of follow-up of the Iranian population
Source: Front Cardiovasc Med. 2023 Jun 9;10:1044638. doi: 10.3389/fcvm.2023.1044638 (PMC10288986; doi:10.3389/fcvm.2023.1044638)
Supplement: Supplementary file 1 [file Table1.docx]

| **Supplementary Table 1.** Comparison of baseline characteristics between respondent and non-respondent groups. | | | | | |
| --- | --- | --- | --- | --- | --- |
|  | | Respondent (N=3685) | Non-respondent (N=1274) | Mean/ Proportion difference (95% CI) | P value |
| Continuous variables, Mean (SD) | |  |  |  |  |
|  | Age (y) | 45.9 (12.7) | 45.3 (11.1) | -0.63(-1.41 to 0.16) | 0.12 |
|  | BMI (Kg/m^2^) | 27.0 (4.7) | 27.7 (4.3) | 0.36(0.05 to 0.68) | **0.02** |
|  | SBP(mm-Hg) | 112.1 (12.2) | 112.0 (12.0) | -0.04 (-0.81 to 0.74) | 0.31 |
|  | DBP(mm-Hg) | 72.8 (8.3) | 73.2 (8.1) | 0.33(-0.19 to 0.86) | 0.82 |
|  | PR(beat/min) | 80.4(11.4) | 80.6(10.9) | 0.26(-0.44 to 0.96) | 0.47 |
|  | PP(mm-Hg) | 39.2(10.2) | 38.9(9.7) | -0.37(-1.01 to 0.28) | 0.26 |
|  | FPG(mmol/l) | 5.5 (1.7) | 5.3 (1.5) | 0.11(-0.22 to 0.0001) | 0.05 |
|  | 2 h-PCPG(mmol/l) | 6.5 (2.9) | 6.5 (2.8) | -0.01(-0.21 to 0.19) | 0.92 |
|  | TC(mmol/L) | 5.0 (1.0) | 5.1 (1.0) | 0.05(-0.01 to 0.12) | 0.12 |
| Categorical variables, n (%) | |  |  |  |  |
|  | Gender (male) | 615 (48.3) | 1556 (42.2) | 0.06(0.03 to 0.09) | **<0.001** |
|  | Hypercholesterolemia | 506 (41.4) | 1609 (43.7) | 0.02 (-0.01 to 0.05) | 0.09 |
|  | Lipid-lowering drugs | 30 (2.4) | 90 (2.4) | 0.001(-0.009 to 0.01) | 0.91 |
|  | Diabetes | 112 (9.2) | 262 (7.1) | -0.02(-0.04 to -0.002) | **0.02** |
|  | Glucose-lowering drugs | 62 (4.9) | 124 (3.4) | -0.01 (-0.03 to -0.002) | **0.01** |
|  | Current smoking | 258 (20.4) | 492 (13.4) | -0.07 (-0.09 to -0.04) | **<0.001** |
|  | Family history of CVD | 221 (17.3) | 684 (18.6) | 0.01(-0.01 to 0.04) | 0.35 |
|  | Low PH/A | 706 (59.5) | 2303 (62.5) | 0.03(-0.002 to 0.06) | 0.06 |
| BMI, body mass index; SBP, systolic blood pressure; DBP, diastolic blood pressure; PR, pulse rate; PP, pulse pressure; FPG, fasting plasma glucose; 2 h-post-challenge plasma, glucose (2 h-PCPG); TC, total cholesterol; PH/A, physical activity | | | | | |

| **Supplementary Table 2.** HRs (95% CI) of CVD for change in BP categories between the baseline visit and first follow-up according to 2017 ACC/AHA hypertension guideline. (N=3685) | | | | |
| --- | --- | --- | --- | --- |
|  | N(event) | Model 1^a^ | | |
|  |  | HR | CI | P value |
| NL to NL | 1672 (92) | 1.00 |  |  |
| NL to elevated | 136 (15) | 1.09 | 0.63-1.90 | 0.75 |
| NL to stage 1 | 374 (36) | 1.43 | 0.97-2.10 | 0.07 |
| NL to stage 2 | 71 (7) | 1.19 | 0.55-2.59 | 0.65 |
| Elevated to NL | 133 (10) | 0.87 | 0.45-1.68 | 0.68 |
| Elevated to elevated | 65 (8) | 1.11 | 0.54-2.30 | 0.77 |
| Elevated to stage 1 | 108 (16) | 1.38 | 0.80-2.37 | 0.24 |
| Elevated to stage 2 | 46 (3) | 0.52 | 0.16-1.64 | 0.26 |
| stage 1 to NL | 312 (26) | 1.46 | 0.95-2.26 | 0.09 |
| stage 1 to elevated | 119 (22) | 2.06 | 1.28-3.30 | 0.003 |
| stage 1 to stage 1 | 417 (61) | 1.93 | 1.39-2.68 | <0.001 |
| stage 1 to stage 2 | 232 (50) | 2.01 | 1.40-2.89 | <0.001 |
| HR, hazard ratios; CI, confidence interval; CVD, cardiovascular disease; BP, blood pressure; NL, normal.  ^a^Model 1: Adjusted for age and gender | | | | |

| **Supplementary Table 3:** HRs (95% CI) of CVD for change in pulse pressure categories between the baseline visit and first follow-up. (N=3685) | | | | | | | |
| --- | --- | --- | --- | --- | --- | --- | --- |
|  | N(event) | Model 1 | |  | Model 2 | |  |
|  |  | HR | CI | P value | HR | CI | P value |
| Q1(<-6) | 878(70) | 1.00 |  |  | 1.00 |  |  |
| Q2 (≥-6 to <0) | 839(61) | 0.88 | 0.63-1.24 | 0.48 | 0.85 | 0.59-1.20 | 0.33 |
| Q3(≥0 to <8) | 1030(108) | 1.30 | 0.96-1.75 | 0.09 | 1.32 | 0.96-1.79 | 0.09 |
| Q4(≥8) | 938(107) | 1.09 | 0.80-1.48 | 0.57 | 1.07 | 0.77-1.48 | 0.68 |
| HR, hazard ratios; CI, confidence interval; CVD, cardiovascular disease; BP, blood pressure; NL, normal.  Model 1: Adjusted for age and gender  Model 2: Adjusted for age, gender, BMI, smoking, education, physical activity, family history of premature cardiovascular disease, hypercholesterolemia, diabetes, baseline SBP, and pulse rate. | | | | | | | |
